# Two-Dimensional Numerical Simulations of Ultrasound in Liquids with Gas Bubble Agglomerates: Examples of Bubbly-Liquid-Type Acoustic Metamaterials (BLAMMs)

**DOI:** 10.3390/s17010173

**Published:** 2017-01-17

**Authors:** Christian Vanhille

**Affiliations:** NANLA, ESCET, Universidad Rey Juan Carlos, 28933 Móstoles, Madrid, Spain; christian.vanhille@urjc.es; Tel.: +34-91-664-74-82

**Keywords:** bubbly liquids, nonlinear acoustics, ultrasound, acoustic metamaterials, filtering, nonlinear frequency mixing, physical acoustics, numerical modeling, bubbly-liquid type acoustic metamaterials

## Abstract

This work deals with a theoretical analysis about the possibility of using linear and nonlinear acoustic properties to modify ultrasound by adding gas bubbles of determined sizes in a liquid. We use a two-dimensional numerical model to evaluate the effect that one and several monodisperse bubble populations confined in restricted areas of a liquid have on ultrasound by calculating their nonlinear interaction. The filtering of an input ultrasonic pulse performed by a net of bubbly-liquid cells is analyzed. The generation of a low-frequency component from a single cell impinged by a two-frequency harmonic wave is also studied. These effects rely on the particular dispersive character of attenuation and nonlinearity of such bubbly fluids, which can be extremely high near bubble resonance. They allow us to observe how gas bubbles can change acoustic signals. Variations of the bubbly medium parameters induce alterations of the effects undergone by ultrasound. Results suggest that acoustic signals can be manipulated by bubbles. This capacity to achieve the modification and control of sound with oscillating gas bubbles introduces the concept of bubbly-liquid-based acoustic metamaterials (BLAMMs).

## 1. Introduction

The use of microbubbles as contrast agents in ultrasound imaging is a well-known technique that has been the topic of important research for decades [1]. The study of acoustic cavitation has been increased during in recent years because of its potential utilization in industrial applications like sonochemistry [2,3]. Sonoluminescence from isolated cavitating bubbles is also of considerable interest [4]. The presence of small gas bubbles in a liquid can be due to boiling, hydrodynamic cavitation, or acoustic cavitation. They can induce benefits or cause drawbacks depending on the context (industry, medicine, environment, etc.). A bubble population can also be intentionally set into a liquid by injecting gas through a porous material or by using contrast agent microbubbles.

The presence of gas bubbles in a liquid greatly modifies its compressibility [2,5,6]. Even at moderate pressure amplitudes, ultrasound becomes nonlinear in bubbly fluids. Moreover, gas bubbles in liquids have already been shown to attenuate acoustic signals propagating in a liquid [2,5,6,7]. In addition, the characteristics of these media are frequency-dependent. The dispersion relation of sound speed, attenuation, and nonlinear parameters depend on the resonance frequency of the bubbles [2,5,6,8]. The medium acquires a very high nonlinear acoustic parameter and a huge attenuation coefficient near the bubble resonance.

Acoustic metamaterials are artificial materials with characteristics that allow the control and manipulation of vibrations and acoustic waves, which are not possible with conventional materials [9]. Research on acoustic metamaterials has been very active lately. New devices designed to have specific absorption properties and stopbands or to allow the guidance of acoustic waves or the acoustic cloaking of different objects are current challenges in the development of acoustic metamaterials.

The unique acoustic characteristics of bubbly fluids make them very interesting in this framework, and the development of devices based on bubbles in liquids constitutes a real and promising challenge [10]. However, the use of bubble populations in liquids to voluntary affect and modify the natural propagation of ultrasound is barely existent in the literature. The manipulation of acoustic waves with bubbles has already been performed, experimentally or numerically, as layers filters [7], as part of an acoustic diode [10,11], as actuators for attenuation and frequency generation of an acoustic diode [12,13], and in the definition of several acoustic switches [13,14].

Our purpose is to study the use of these dispersive properties to control sound and to set out the development of bubbly-liquid-type acoustic metamaterials (BLAMMs). We focus our attention on the attenuation and nonlinear acoustic effects of bubbles. Filtering properties and nonlinear frequency mixing are theoretically analyzed by considering different monodisperse bubble populations confined in restricted areas of liquid to evaluate the possible manipulation and control of ultrasound with bubbles of determined sizes.

In this paper, we use the sensitivity to frequency, due to dispersion, at moderate pressure amplitudes to modify the frequency composition of ultrasonic waves in a liquid. Two-dimensional numerical simulations are performed to evaluate the effect of bubbles on ultrasound by calculating their nonlinear interaction. The methodology relies on the high nonlinear acoustic parameter and high attenuation coefficient that exist in a small frequency range around the bubble resonance. The first method consists in the use of bubbles in a restricted zone to withdraw frequencies at their resonance from a pulse. The frequency values retrieved from the signal can be controlled by modifying the bubble sizes. The second technique explores the generation of a low ultrasonic frequency component from the nonlinear mixing of two high ultrasonic harmonic signals traveling within a small area with resonant bubbles. The high nonlinearity of the medium at bubble resonance allows us to carry out this process at rather small amplitudes. The creation of this frequency component can be controlled by the bubble size and the gap used between the two primary frequency values. Both methods are tested in water with air bubbles.

The results obtained show the effects that bubbles have on acoustic waves and evidence their potential for use in the development of acoustic metamaterials. This work suggests that these simple processes could be used in the future to manipulate sound in the technological, industrial, or medical contexts.

In what follows, Section 2 briefly presents the differential model used to perform the numerical simulations described in Section 3, and Section 4 discusses the results to show the potential of using bubbly media with dispersive attenuation and nonlinearity to act on the frequency composition of ultrasound.

## 2. Materials and Methods

**Figure 1:**
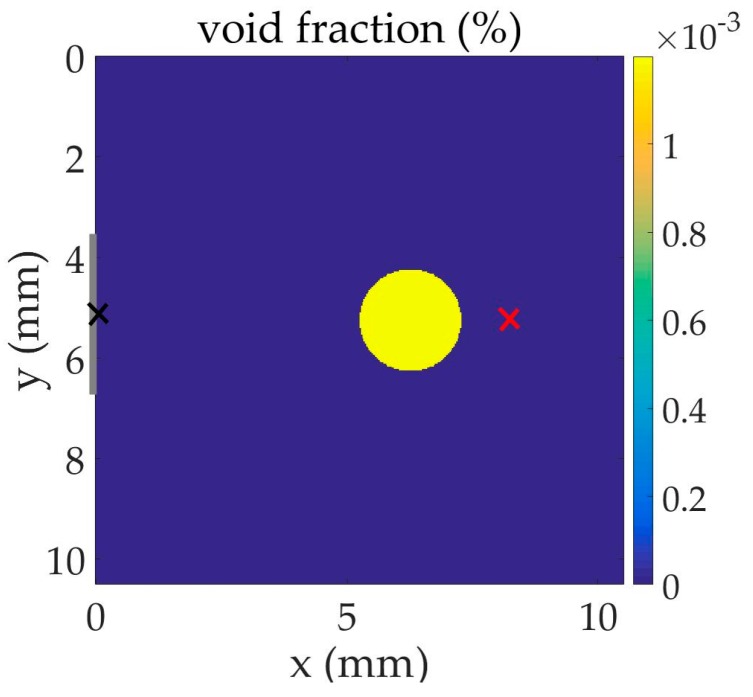
Schematic representation of the two-dimensional problem in which the black and red crosses represent the input and output signal positions, respectively.

We consider a population of small gas bubbles present in a volume of liquid in which ultrasound travels. The two-dimensional schematic representation of this problem is shown in Figure 1.

The liquid, assumed to be linear, inviscid, and nondispersive, is excited by the ultrasonic source placed at x=0  m. The propagation of the mechanical wave is modeled by the linear wave equation of second order written in acoustic pressure variable, *p*, Equation (1). The oscillations of bubbles submitted to the acoustic field are modeled by a Rayleigh–Plesset equation, written here in terms of the bubble volume variation variable, *v*, Equation (2). The coupling of both equations models the nonlinear interaction of both the acoustic and bubble oscillation fields [15]:

∇^2^*p* − *p*_tt_/c^2^ = −ρN*v*_tt_, 0 < x < L, 0 < y < l, 0 < t < T, (1)

(2) $$v_{tt}+\delta \omega v_{t}+\omega^{2}v=av^{2}+2bvv_{tt}+bv_{t}^{2}-\eta p\;,\;0\leq x\leq L,\;0\leq y\leq l,\;0<t<T.$$

In these equations, variables *p* and *v* are functions of the two-dimensional space coordinates (x,y) and time t, subscripts indicate derivatives, T is the last instant of the study, L and l are the dimensions of the space domain of the study in directions x and y, $\nabla^{2}$ is the Laplacian operator, c and ρ are the sound speed and density of the liquid, N is the density of bubbles of initial radius R and volume V considered in the liquid, resonant at $\omega_{r}=2\pi f_{r}$, δ is the viscous damping coefficient of the bubbly fluid, η=4πR/ρ, a = ω^2^(γ + 1)/(2V), b=1/(6V), and γ is the specific heats ratio of the gas. Since the nonlinear parameter of the bubbly liquid is much higher than the nonlinear parameter of the homogeneous liquid [2,5,6], the model assumes that the nonlinearity that affects the system is due to the presence of bubbles only. In this differential system the linear pressure equation is coupled to the nonlinear bubble equation through the last term of each equation. This nonlinear coupling mechanism turns into the formation of amplitude-dependent nonlinear distortion of both the pressure field and the bubble vibrations, and produces dispersion and attenuation of the pressure wave. The sound speed is strongly frequency-dependent, as are the attenuation and the compressibility, and the nonlinear parameter, in the bubbly medium. The very high value of the nonlinear parameter makes it possible to produce nonlinear effects (harmonic generation by nonlinear distortion, difference frequency creation by nonlinear frequency mixing) using rather low finite pressure amplitudes. Thus, when a finite-amplitude ultrasonic wave interacts with a population of small oscillating gas bubbles that are present in a reduced region of the two-dimensional space, these bubbles strongly affect the ultrasound.

Before the onset of the source, the bubbles are assumed to be without oscillation, and no mechanical wave is considered in the liquid. Once the source placed at x=0 starts exciting the fluid at the angular frequency $\omega_{1}=2\pi f_{1}$ (harmonic signal or pulse), and eventually in combination with the angular frequency $\omega_{2}=2\pi f_{2}$, and with the pressure amplitude $p_{0}$, an acoustic wave propagates in the fluid. The time-dependent distribution along y at the source face is defined by $g_{s}\left(y,\omega_{1}\;t,\omega_{2}\;t\right)$. Open-field conditions are imposed at x=L, y=0, and y=l through the non-reflecting pressure functions $g_{L}\left(y,t\right):\;p_{x}=-p_{t}/c$, $g_{0}\left(x,t\right):\;p_{y}=p_{t}/c$, and $g_{l}\left(x,t\right):\;p_{y}=-p_{t}/c$, respectively, where subscripts indicate derivatives. These considerations lead to the following initial and boundary conditions associated to the differential Equations (1) and (2):

(3) $$\begin{array}{l} p\left(t=0\right)=p_{t}\left(t=0\right)=0,\;0<x\leq L,\;0\leq y\leq l,\\ v\left(t=0\right)=v_{t}\left(t=0\right)=0,\;0\leq x\leq L,\;0\leq y\leq l,\\ p\left(x=0\right)=p_{0}\;g_{s}\left(y,\omega_{1}\;t,\omega_{2}\;t\right),\;0\leq y\leq l,\;0\leq t\leq T,\\ p_{x}\left(x=L\right)=g_{L}\left(y,t\right),\;0\leq y\leq l,\;0<t<T,\\ p_{y}\left(y=0\right)=g_{0}\left(x,t\right),\;0<x\leq L,\;0<t<T,\\ p_{y}\left(y=l\right)=g_{l}\left(x,t\right),\;0<x\leq L,\;0<t<T. \end{array}$$

Further information concerning the mathematical model and its physical hypothesis and restrictions, and the numerical model Snow-Bl used to solve the problem can be found in [15]. It is worth noting that acoustic cavitation, bubble collapse, buoyancy, Bjerknes forces, and acoustic streaming are not considered in the numerical simulations presented in this work.

## 3. Results

Two different experiments are performed in Section 3.1 and Section 3.2. In each configuration assumed here, one or several discs of liquid of radius 1.01 mm in which gas bubbles are present are considered in the liquid volume. Each disc represents a three-dimensional cylinder in a more realistic configuration that approximates a column of a mixing of liquid and gas bubbles in a three-dimensional liquid volume. An ultrasonic signal propagates in the liquid and interacts with the bubble disc. The output of the system (signal measured at a hydrophone) is compared to the input of the system (signal emitted from a pulse transducer or a two-frequency transducer) when bubble cells are placed in between. At x=0  mm, the time-dependent plane source distribution along y, shown in grey in figures, takes the central part of the face over 3.39 mm with two short smooth transitions at its edges to avoid discontinuities. The centers of the bubble cells, source, and receiver are on the symmetry axis of the problem.

### 3.1. Filtering Specific Frequencies from an Ultrasound Pulse through a Bubbly-Liquid Cell Net

The schematic representation of the two-dimensional problem is shown in Figure 1. Water and air are used in the experiment: $c=1500\;m\cdot s^{-1}$, $\rho =1000\;kg\cdot m^{-3}$, and γ=1.4. Bubbles resonant at $f_{r}=2\;MHz$ (R=1.68  μm) are concentrated in the cell centered at x=6.26  mm on the symmetry axis with the density $N=6\times 10^{11}\;m^{-3}$. The sound speed at $f_{r}$ drops from 1500 to 1341 m·s^−1^ when bubbles are added, whereas the global density is not significantly changed. The viscous damping in the bubbly fluid is δ=0.16. The dispersion curve for attenuation coefficient of this bubbly fluid, calculated from the application of an analytic perturbation method [2,5], shows a high peak value at bubble resonance.

**Figure 2:**
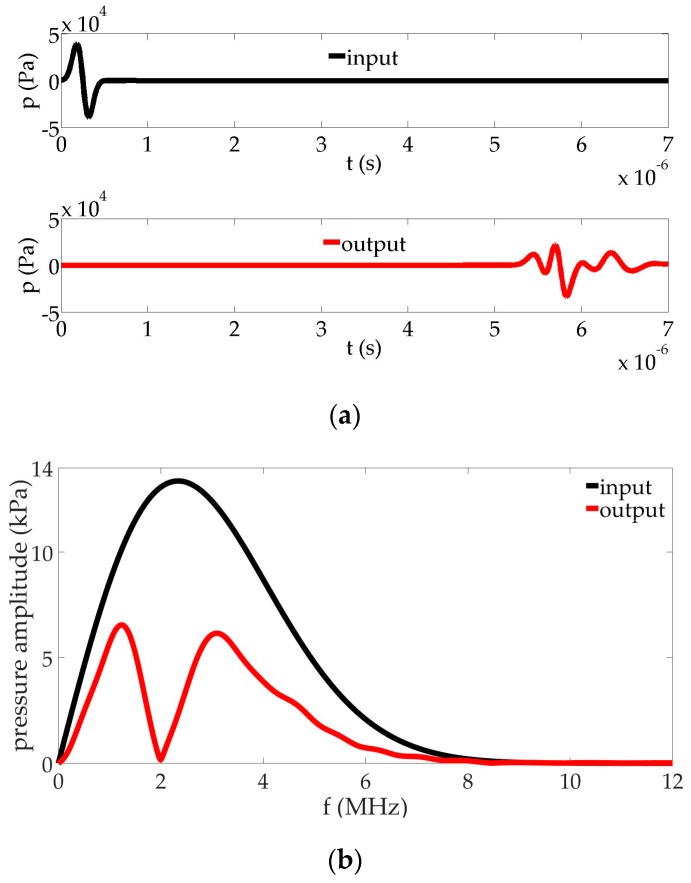
Comparison of input and output signals in the configuration given in Figure 1: (**a**) waveform; (**b**) frequency content.

A large-band ultrasonic pulse is used at the source (see Figure 2b, black line). The wave propagates in the fluid and interacts nonlinearly with the bubbles contained in the cell. We study the capacity of the cell to filter part of the frequencies of the pulse. Figure 2 analyzes the response of the system by comparing the waveforms and frequency content of the pulse at the source (input, black cross on the symmetry axis) and beyond the bubbly-liquid cells (output, red cross at x=7.90  mm on the symmetry axis). Since an important amount of energy is lost during the spreading of the wave from the source into the liquid (which is not a plane wave) and due to the impedance change at the interface between the liquid and the bubbly-fluid cell, the entire pulse is lowered in amplitude at the output. The time evolution indicates a strong distortion of the pulse after traveling through the bubble cloud. In addition, the output signal clearly shows that the bubbly cell cancels the propagation of the frequency component at the bubble resonance. The cell acts as a frequency filter.

**Figure 3:**
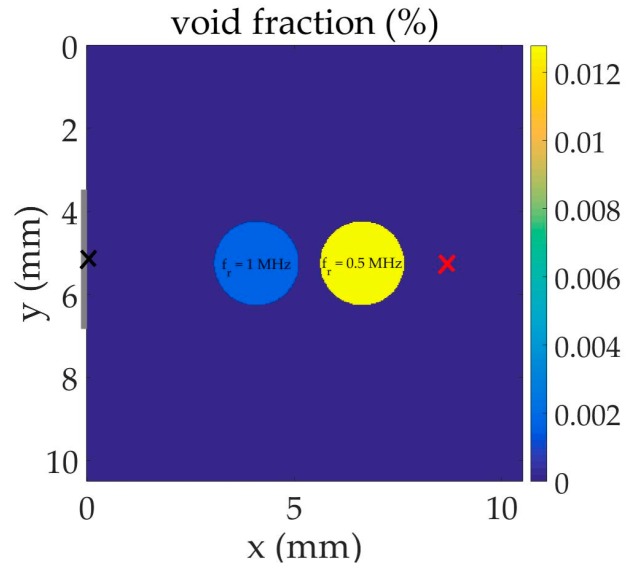
Schematic representation of the two-dimensional problem in which the black and red crosses represent the input and output signal positions, respectively.

**Figure 4:**
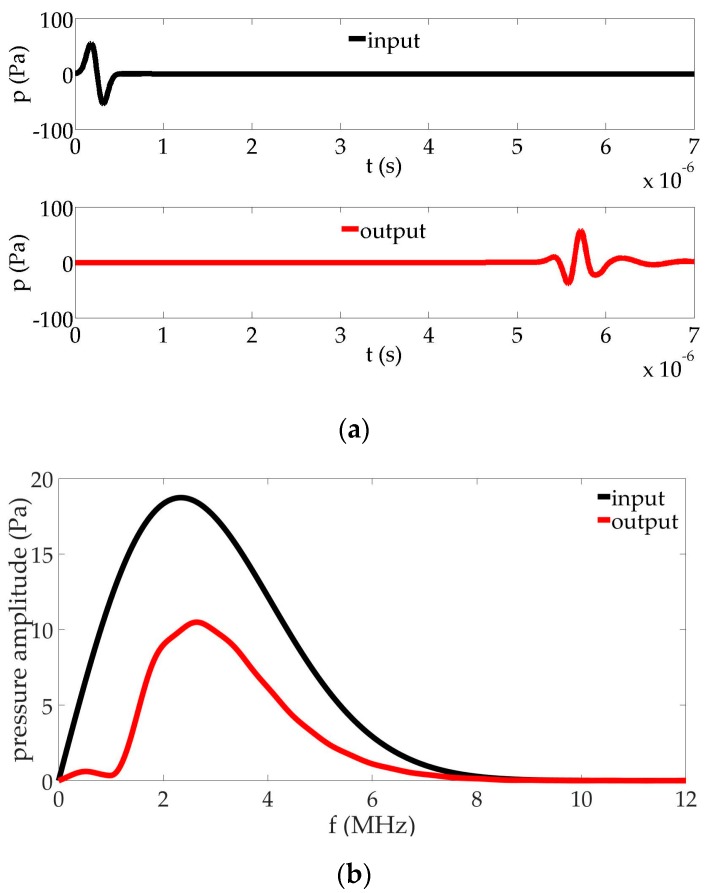
Comparison of input and output signals in the configuration given in Figure 3: (**a**) waveform; (**b**) frequency content.

A net of two bubbly cells centered at x=4.08  mm and x=6.63  mm on the symmetry axis is now considered in the liquid as shown in Figure 3. Air bubbles resonant at f_r_ = 1 MHz (R=3.37  μm) and f_r_ = 0.5 MHz (R=6.73  μm) are concentrated in the cells with the density $N=1\times 10^{11}\;m^{-3}$. The sound speed in the bubbly cells at $f_{r}$ drops to 1040 m·s^−1^ and 303 m·s^−1^, respectively. The viscous damping in the bubbly fluids are δ=0.08 and δ=0.04, respectively. The dispersion curves for attenuation coefficient of these bubbly fluids show a high peak value at their respective bubble resonance. Figure 4 compares the pulse in the time and frequency domains at the source (input, black cross) and beyond the bubbly-liquid cells (output, red cross). In this case, in addition to the strong distortion of the pulse, the output signal shows that a large stop band around the bubble resonances is obtained. The capacity of the cells to annihilate the propagation at specific frequencies is clear. It must be noted that, without the 0.5 MHz bubble region, the output spectrum presents a profile quite similar to the one of Figure 2, showing the cancellation of the propagation at 1 MHz and larger amplitudes around 0.5 MHz.

No modifications in the filtering effects have been observed by changing pressure amplitudes, i.e., the nonlinearity does not have a preponderant effect on filtering in this case. These experiments suggest that the filtering of part of an ultrasonic pulse can be performed by using small cells with mixtures of liquid and gas bubbles. This means that many different possibilities of filters could be created and used to manipulate ultrasonic signals with bubbles.

### 3.2. Generation of Specific Frequencies from a Two-Frequency Ultrasound Signal through a Bubbly-Liquid Cell

**Figure 5:**
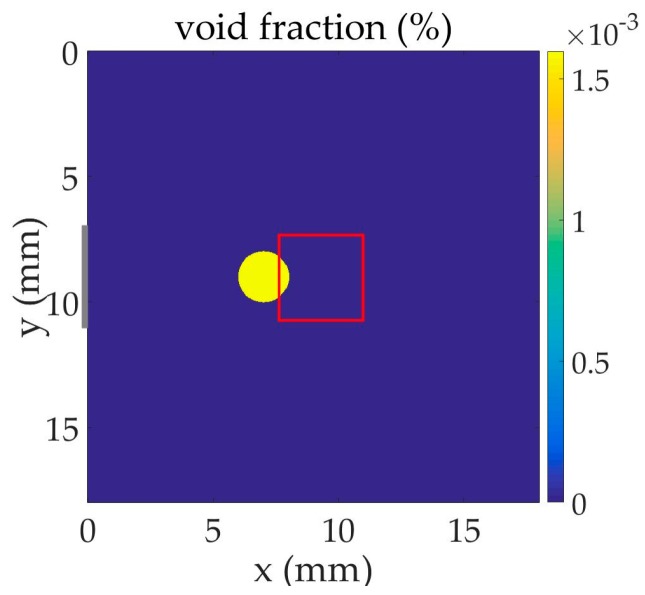
Schematic representation of the two-dimensional problem in which the red square represents the output signal area displayed in Figure 6.

The schematic representation of the two-dimensional problem is shown in Figure 5. Water and air are used in the experiment: $c=1500\;m\cdot s^{-1}$, $\rho =1000\;kg\cdot m^{-3}$, and γ=1.4. Bubbles resonant at $f_{r}=1\;MHz$ (R=3.37  μm) are concentrated in the disc centered at x=7.02  mm on the symmetry axis with the density N = 1 × 10^11^ m^−3^. The sound speed at $f_{r}$ drops from 1500 to 1040 m·s^−1^ when bubbles are added, whereas the global density is not significantly changed. The viscous damping in the bubbly fluid is δ=0.08. The dispersion curve for compressibility of this bubbly fluid indicates a high peak value at bubble resonance.

**Figure 6:**
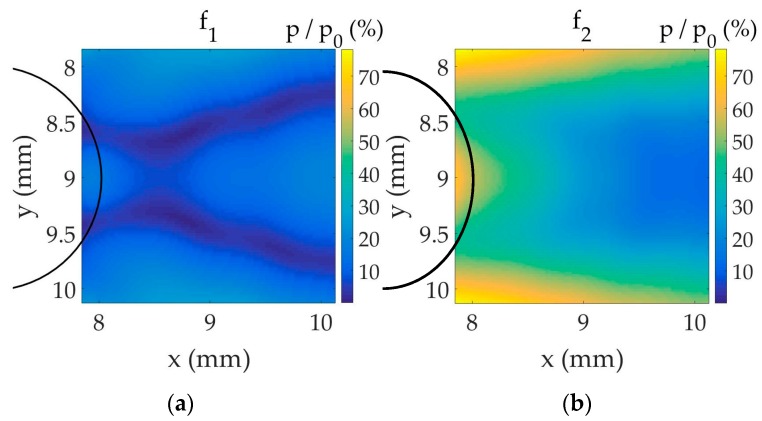

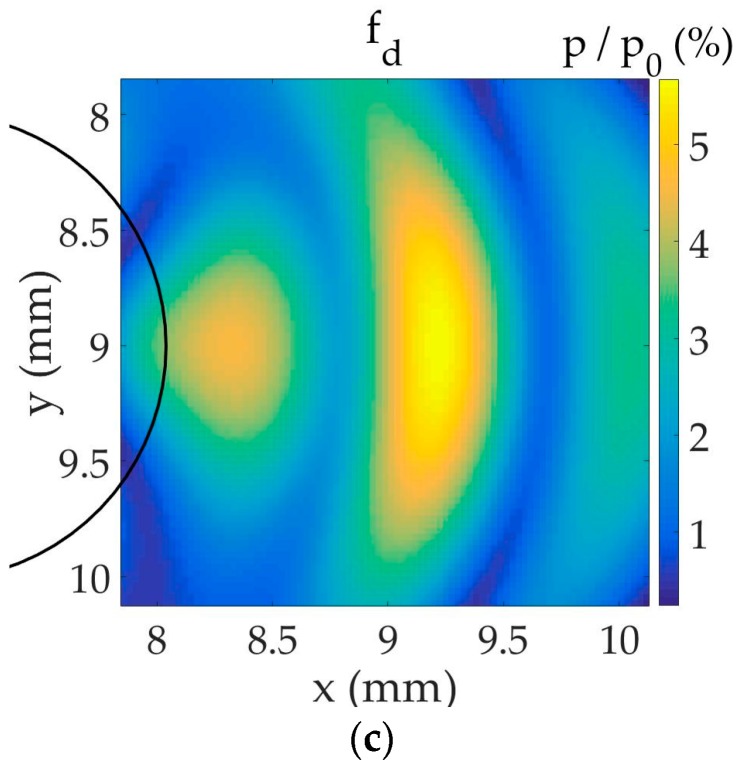
Amplitude distribution, expressed in % of pressure at the source, of the two primary frequencies (for which the same color range is used) and the difference frequency (for which a specific color range is used) within the squared area shown in Figure 5. The bubble region boundary is shown with the black line. (**a**) $f_{1}$; (**b**) $f_{2}$; (**c**) $f_{d}$.

A combination of two harmonic signals at frequencies $f_{1}=f_{r}=1\;MHz$ (angular frequency $\omega_{1}=2\pi f_{1}$) and $f_{2}=1.25\;MHz$ (angular frequency $\omega_{2}=2\pi f_{2}$) excites the fluid with the pressure amplitude $p_{0}=5\;kPa$. An ultrasonic wave propagates in the fluid and impinges the bubbly-fluid cell. Bubbles and pressure wave interact nonlinearly. We study the generation of the difference frequency $f_{d}=250\;kHz$ by nonlinear mixing (parametric arrays) of the two initial signals (input) through the bubbly-liquid disc by analyzing the amplitude $p_{d}$ of this component $f_{d}$ beyond this disc in a squared area shown in Figure 5 (output, red square). The result is represented in Figure 6 (relative amplitude values of primary frequencies, $f_{1}$ and $f_{2}$, and difference frequency, $f_{d}$, in relation to pressure amplitude at the source, $p_{0}$). Beyond the bubbly cell, the primary frequencies do not present a peak, whereas the difference frequency attains a peak. The maximal $p_{d}$ value is reached at the point x=9.22  mm on the symmetry axis in this case. Even if the values (lower than 6% of $p_{0}$) obtained here are much lower than in resonators [16], the response of the system in terms of difference-frequency amplitude is clear and shows that the area of maximal values is situated beyond the cell from the source. The strong attenuation of $f_{1}$ in this area and the existence of a smaller secondary $p_{d}$ peak very near the bubble region boundary, around x=8.3  mm on the symmetry axis, must be noted.

This experiment suggests that low ultrasonic signal can be generated by varying the bubbles in the cell or by adding cells with bubbles of other sizes. This means that many different signals could be created and used beyond a bubbly-fluid cell net.

## 4. Discussion

Two methods for modifying the frequency composition of ultrasound by using bubble aggregates have been tested in Section 3. The results obtained from the numerical simulations show the effects that bubbles have on acoustic waves and evidence their potential for use in acoustic metamaterials. This work suggests that these simple processes could be used in the future to manipulate sound in technological, industrial, or medical contexts.

Section 3.1 presents a process that suggests the possible development of a device formed by bubbly cells that might be able to remove from an acoustic signal certain frequencies or frequency bands that are unwanted, while the others remain in the signal. This signal processing could be employed to suppress noise or interfering frequencies with other devices, humans, and animals.

According to [17], this device could be applied for filtering frequency components potentially harmful for some animal species under some given conditions (natural accidents, explosions). For example, the use of air bubbles can protect whales during controlled explosion in off-shore gas.

Section 3.2 presents a process that suggests the possible development of a device formed by bubbly cells that is based on the nonlinear frequency mixing to generate lower frequency components [18]. This process could be employed in applications that require the propagation of directive signals with lower dissipation in applications such as non-destructive testing and imaging [19].

The results shown in Section 3 might be employed for the sizing of bubbles aggregated in an area of the liquid. A large-band pulse used in a procedure similar to Section 3.1 could detect the resonance of bubbles by attenuation and thus deduce their size. The measurement of the maximal difference-frequency amplitudes beyond a bubble cloud using a procedure similar to Section 3.2 by sweeping the primary frequency $f_{1}$ could detect the bubble resonance by nonlinearity and thus could sense the bubble size. This process could be used to characterize acoustic cavitation activity [20,21,22].

In this sense, in situations in which a given event produces bubbles in a liquid (explosion, hydrodynamic cavitation, acoustic cavitation, gasification in nuclear plant coolant, animal behavior, or natural accident), the above considerations could help provide information about the size of the resonant bubbles and thus could inform somehow about the kind of event that gave rise to the bubbles and contribute to the recognition of this event.

Finally, we can imagine a more complex BLAMM device that uses a bubbly-liquid cell that is able to create new frequencies from several couples of primary frequency signals, $f_{1}$ and $f_{2}$, of moderate amplitude (Section 3.2), and that then performs a selection of one or some of these frequencies through their annihilation by using other different bubbly-liquid cells (Section 3.1).

The effects undergone by ultrasound are sensible to variations of the bubbly medium parameters. These modifications induce important alterations of the frequency components and amplitudes of ultrasound. The sensitivity of both processes shown in Section 3 to variations of the physical parameters must be tested. Future research will have to be performed by varying the bubble density, the size and the geometry of cells, and by diversifying their number and locations.

In Section 3, the methods are presented for bubbles of a single size in each cell (monodisperse bubble population). Their extrapolation can be imagined for the detection of bubbles of different sizes in the same liquid medium.

It must be noted that some authors reported results that, even if they were obtained in different configurations, roughly coincide with the results presented here when bubbles are present in a liquid. The strong reduction of the magnitudes at the primary frequencies due to the high attenuation of the microbubbles and the strong enhancement of the magnitude of the difference frequency because of the bubble resonance was mentioned in [23]. A very important gap between the amplitudes of the primary and difference frequencies was reported in [22,24]. Experimental works underpinned by this theoretical study will have to be performed.
